# The appearance of discretionary income: Influence on the prevalence of under- and over-nutrition

**DOI:** 10.1186/1475-9276-4-10

**Published:** 2005-06-28

**Authors:** Robert J Karp, Cindy Cheng, Alan F Meyers

**Affiliations:** 1 Department of Pediatrics. SUNY-Downstate Medical Center 450 Clarkson Ave. Brooklyn, NY. 11203 USA; 2 Department of Pediatrics. SUNY-Downstate Medical Center 450 Clarkson Ave. Brooklyn, NY. 11203 USA; 3 Department of Pediatrics. Boston Medical Center 260 Franklin St., Boston, MA 02110. USA

## Abstract

Undernutrition – protein energy malnutrition or specific nutrient deficiencies – has been an inherent characteristic of impoverished populations throughout the world. Over-nutrition, obesity and nutrition imbalance is a current concern among those with rising though still insufficient incomes. We review data to suggest that the prevalence of these forms of malnutrition in populations is highly influenced by the rate of appearance of discretionary income.

In developed countries, discretionary (alternatively "disposable") income refers to funds available after obligate payments (rent, heat, and the cost of getting to work) and payment for necessities (food and clothing). For families living at or below poverty, the last dollar earned is spent on these obligations. Undernutrition is common. By contrast, likelihood for obesity or imbalance increases with rising income when that last dollar is earned without certainty that it is available for discretionary spending. In the United States, neither under- nor over-nutrition is likely when new income is free and clear of debt or obligation. This occurs at approximately three times the poverty level.

While income poverty and food insecurity affect *risk *for malnutrition rather than *outcome*, nutrition education programs that address issues of income and food support increase likelihood for adherence to recommendations.

## Introduction

Macrosocial phenomena (food costs and food culture), distal from the life of family and child, affect the prevalence of poverty related malnutrition. [1-4] Which specific families are affected, however, is highly dependent on micro-social factors in the family related to how well resources are used in the proximal or microsocial environment. [1,4,5] For the casual observer, failure to nurture and nourish children within the microsocial environment characteristic of poverty is seen as the *cause *rather than the *consequence *of poverty. [6-8] This view fails to recognize that for the poor, behavior is shaped by the power of a macrosocial environment they are unable to change, distal from the experiences of children and family. [6] Choices that seem irrational or even self-destructive from the perspective of those living outside such a powerful environment may seem perfectly appropriate and effective from within. [4,6,7]

We suggest that in the United States and other developed countries, a lack of discretionary income affects food choices and ultimately nutritional status. Untoward outcomes include undernutrition – growth retardation or specific nutrient deficiencies, and overnutrition – primarily obesity. [9] The specific *economic *influence on nutritional status is the appearance of discretionary income. Neither under- nor overnutrition are common when new income is free and clear of debt or obligation – past three times the poverty level. The prevalence of these forms of malnutrition in the United States and other developed societies is highly influenced by both the level of income and support and the availability of discretionary income. The essential question is "How will the last dollar earned be spent?"

## Non-economic elements have a profound influence on nutritional status

Economic factors, taken alone, do not determine the nutritional status of individual children in a family or community. [5,10] Maternal depression, for example, is a critical factor affecting parenting ability, child nutrition and development. [11-13] Parental beliefs and practices have a profound effect on the quantity and variety of food that is brought into the family as well as the allocation to each member. [14,15] As Gopalan writes,

"Differences in the nature of intra-familial distribution of food, in particular in infant feeding and childrearing practices, between the families and between communities can result in important differences with nutritional status (especially of children) between households, and between communities with nearly similar overall levels of dietary inadequacy." [14]

Moreover, as often found in immigrant populations, poverty in the past may affect present-day food selection, [16] and the poor in the United States are surrounded by the wealthy, and the world of wealth presented via television and other mass media begs to be imitated. In 1943, Margaret Mead, with great prescience, warned of the consequences of having the poor imitate the affluent. She wrote,

" [While] an arbitrary balanced diet would be superior to the meals habitually eaten by the worst fed third of our population, there is a danger that the conventions of a balanced diet may sift down as 'style' to the lower income levels without the necessary knowledge to see that the meal is really balanced." [17]

Adjustments to chronic poverty seem to establish cultural norms and may appear to be separate from their economic origins, but they are not. [18] Food preferences formed in childhood last a lifetime [10,19] and affect the likelihood of malnutrition occurring. Thus, in the context of chronic poverty, what we see as "food choice" is a highly complex phenomenon influenced by the cost and availability of food and the dynamics of the family. On a positive note, the body of Mead's work, [17] and that of her contemporaries [9,19,20] suggest that a combination of education, persistence, readily available, affordable and culturally consistent food is likely to have a positive impact on the nutritional status of children in poor families. It is necessary, however, to have a secure food supply. [21]

## The impact of poverty on nutritional status

With chronic poverty, a process called the "Engel's Phenomenon" occurs. Food selection narrows to those items providing the most energy at lowest cost. Over time, micronutrients disappear from the diet, and specific nutrient deficiencies follow. [22,23] For families living well below the poverty level, increasing income does increase discretionary spending. Any money received is used to pay the cost of necessities – food, rent, heat, and the expenses of getting to work such as clothes, day-care, and transportation. [2,3] Nothing is left to improve nutritional quality of the diet or, at lowest income, provide sufficient caloric intake. Thus, there is a paradoxical association between poverty and obesity in childhood. As shown by Hofferth and Curtin with data from Panel Study of Income Dynamics Child Development Supplement of 1999, leanness is the rule (a 12% incidence of obesity) for families living below poverty level. (24) Twenty two percent of children living in families with incomes from one to three times poverty level are likely to be obese. Past three times poverty income, the incidence among children diminishes to the 12 % level again. An important difference, however, is that impoverishment increases the risk for micronutrient deficiency. [1,22]

Studies throughout the United States show that competing costs for other necessities – rent [25] and fuel [3] reproduce the "Engel's Phenomenon" described above. In the figure below, an "Engel curve, " poverty level is defined as three times the cost of the United States Department of Agriculture's Low Cost Food Plan (USDA-LCFP). [26] Theoretically, at the poverty level, the total income goes to pay the obligate expenses and the cost of necessities – 1/3 for food, 1/3 for housing, and 1/3 for other necessary expenses. Only past poverty incomes does discretionary income appear where discretionary income equals total income minus the cost of obligate expenses and necessities. [26]

**Figure 1:**
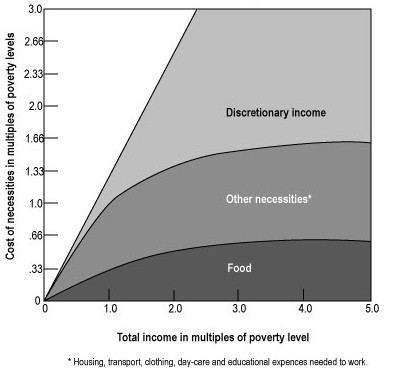
Engel Curves. Poorer workers' increased earnings do not generate discretionary income until the total of income from earnings plus supplementation reaches poverty level. Past poverty level discretionary income appears until, at about three times poverty level income, all new money earned is available for discretionary spending. – not necessarily food, housing or other essential needs. (This figure is reproduced from Malnourished Children in the United States with permission).

Discretionary (alternatively "disposable") income refers to funds available after obtaining necessities. These costs include food, housing, health care and the expenses of maintaining employment – day care, clothes and transportation. The interaction between definitions of poverty and sources of income and resources is fully described by Citro and Michael. [26] (Figure 1).

There is a curvilinear relationship between poverty and childhood obesity. [24,27] At poverty level or below, all income goes for necessities. Increasing the total income has no effect on discretionary income since none accumulates. Children in families with incomes below the poverty are most 'at risk' for undernutrition – either growth retardation or specific nutrient deficiency. From 1 to 3 times poverty level incomes, discretionary income appears until, at three times the poverty level, all new income is discretionary. As the data provided by Hofferth and Curtin suggest, children who live in families with incomes at the margin of poverty and sufficiency are at increased risk for obesity. [24] These data reinforce similar observations by Garn in his evaluation of data from the Pre School and Ten State Nutrition Surveys of 1968–70. [27] Leanness was found among the poorest and most affluent while adults living in mid-level income families were more likely to be obese.

The ratio of change in food expenditures to change in income is called the *marginal propensity to spend on food*. (5) In developing countries, the marginal propensity to spend on food is high for the poor and low for the affluent. Similarly, in the United States, the last dollar earned by a poor family will represent obligate spending with some part going for food. For the affluent, expenditures for food change little with increased or decreased income. Rather, increased income provides an opportunity for choice as new income is available entirely for discretionary spending. This "income of opportunity" is not available to the poor.

Thus, the appearance of discretionary income will have a profound effect on risk for malnutrition. It is a marker for the *opportunity *to be "plump and healthy." Low cost, high-energy foods are readily available and even desirable for families at-risk for food insecurity. [28] Once sufficient income is obtained, concern for the cost of necessities recedes, and neither under nor over nutrition is likely. The opportunity to be "plump and healthy" can be rejected.

A final consideration, deriving from the work of David Barker and colleagues in England [29] is that undernutrition in-utero and during the first months of life is a precursor of obesity and the elements of the metabolic syndrome later in life. Other data suggest that the obese mother with or without gestational diabetes mellitus is likely to have a child prone to metabolic syndrome. [30] The impact of food security on transgenerational obesity is yet to be studied.

## Are these comments applicable internationally?

These observations provide a conceptual framework for what is now seen in economically developed countries. An oft seen phenomenon in urban populations in developing countries is the coexistence of a "double burden" of under and overnutrition in families with rising incomes. [31,32] As some parts of the population move from poverty to relative affluence, they are likely to become obese, diabetic, hypertensive and be affected by atherogenic cardiovascular disease the dysmetabolic syndrome. [32,33]

**Figure 2:**
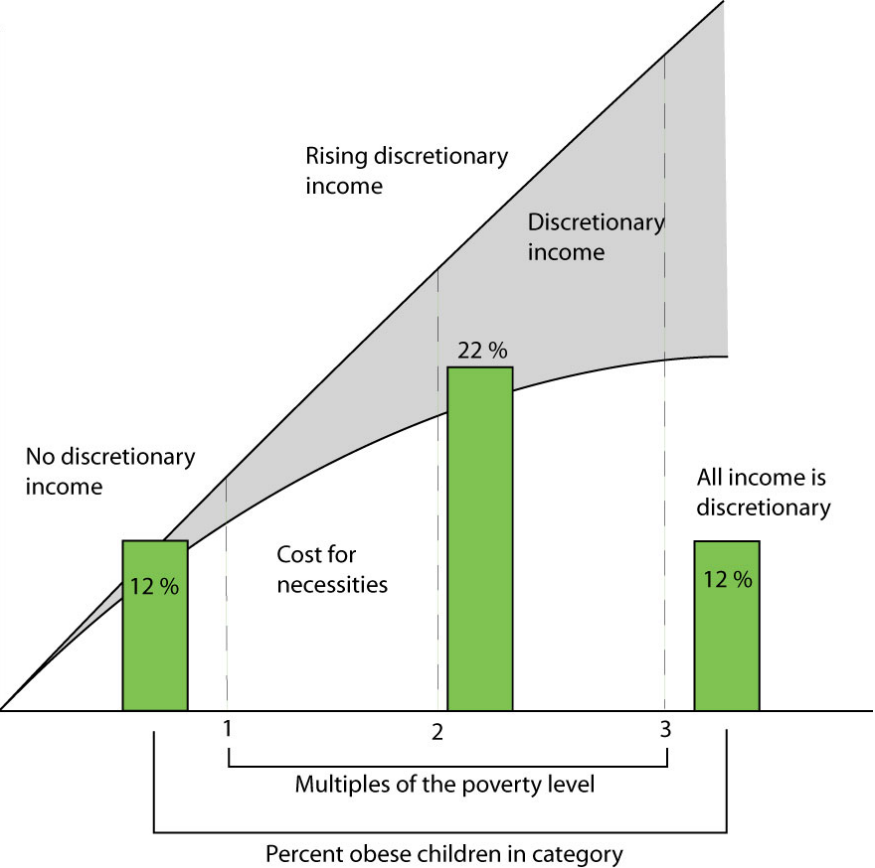
The movement from wide-spread poverty ("developing") to affluence ("developed") is likely to be accompanied by a middle phase where incomes rise, but the population does not feel secure. Without systems of support, the likelihood of overnutrition and nutrient imbalance will increase.

As shown in Figure 2, the movement of subpopulations through economic levels does not go directly from undernutrition to optimal nutrition. Unless steps are taken to assure affordable foods, rising incomes are likely to be associated with obesity, imbalance and persistent specific nutrient deficiency. (Figure 2)

The model presented herein is applicable in developed countries other than the United States. As the indigenous populations of industrial democracies age and diminish in size, it is possible that resident aliens, those people needed to provide essential services, will not receive the same entitlement to health and nutritional benefits as the citizen. The model used in the United States, where the existence of substantial numbers of chronically poor people is an accepted norm, may spread to other developed economies.

## Effective interventions for physicians and other health workers

Two interacting sets of interventions are available to health professionals. These are, first, addressing the impact of income poverty on food selection and second, providing support for use of affordable, healthful foods. The model for providing these interventions is the "Medical Home." As described by Calvin Sia, pediatricians should see themselves as part of a team in which their unique part is providing well and sick care. [33] The over-all milieu, however, should have elements necessary to provide access to care and resources, outreach to the community and elements of advocacy for unmet needs. Thus, the macro-social issues raised here are appropriate to pediatric care.

## The importance of addressing income poverty and its effect on food resources

A general recommendation for the sake of a healthy society is to follow the example of other industrial democracies – take advantage of the wealth generated by a free-market economy to maintain the general well being – including good nutrition – of our people. [34] Successful interventions include earned income tax credits, supplemental programs for food, housing, day-care, transportation, health care, and at times, direct income subsidies. [35]

With respect to food costs and availability, supplemental programs (WIC and Food Stamps) are particularly effective in maintaining high quality diets. These improve nutritional status in two ways. First, the foods given have a high nutritional value. Second, families spend the same dollar amount to meet less energy needs. Thus, the nutrient value of the foods purchased by a poor family increases to that of "non-poor" essentially reversing the Engel's phenomenon. Supplemental food programs have shown substantial impact on nutrition in pregnancy and limit weight gain for older children and adults. A study of low-income households, both food secure and insecure, revealed that food assistance programs lower the risk of overweight in low-income children, particularly girls in food insecure households. As the late Jean Mayer has written, " [P]opulations which expect to be subjected at regular intervals to scarcity of food may consider a certain measure of obesity as desirable, indeed as necessary for survival." [16]

## Assessing for food insecurity

A simple addition to a screening evaluation is to assess income expenses, resources for food and food insecurity. The first three questions of the USDA/Cornell Radimer food insecurity surveys are used in our work to determine which children are "at-risk." [36] (Table 1).

**Table 1:** Is this child "at-risk" for food insecurity?

Please tell me whether the following statements are true of you:
A. I worry whether my food will run out before I get money to buy more. Often true [_]; Sometimes true [_]; Never true [_]
B. I worry about whether the food I can afford to buy for my family will be enough. Often true [_]; Sometimes true [_]; Never true [_]
C. (Only for people who give a positive answer to A or B). The food I buy just doesn't last, and I don't have money to get more. Often true [_]; Sometimes true [_]; Never true [_]

These questions are adapted from the CDC and Cornell/Radimer Food Insecurity Questionnaires. [36] They are highly sensitive and likely to capture all children affected by food insecurity or hunger.

## The importance of addressing food culture and nurturing of children

It is necessary in pediatric practice to identify the impact of convenience food use. A study in a poor community in north Philadelphia, compared families with malnourished children, to families with well nourished children. [37] Each family was presented with three lists of foods: those requiring preparation by an adult called "basic," those not requiring home preparation but nutritious called "healthy," and those not requiring preparation but having a low nutrient to energy content called "non-nutritious." The essential difference between family groups was that families of malnourished children were dependent on convenience food. The cost for energy of the "healthy" foods was substantially higher than that for the non-nutritious. Other differences were found in knowledge and behavior of parents.

The use and misuse of convenience foods, characteristic of contemporary food consumption patterns in the United States, illustrates how economic and social factors interact to affect nutritional status among the poor. Cheaper convenience foods have lower nutrient density (high fat or sugar content) compared with nutritious ones which people with higher incomes can afford to purchase. [22] A cereal grain, legume and scant meat diet, as recommended by the LCFP-USDA can be obtained within the allotment, but to achieve savings, families require an adult in a home with resources and ability to prepare food and a place to obtain affordable food staples. [34] One cannot assume, however, that these conditions exist in poverty level families. The need, therefore, is to develop model programs capable of increasing awareness and knowledge. [4] The characteristics of successful programs are listed in Table 2

**Table 2:** Increasing quality of nutrition education in "at-risk" communities

Successful programs for nutrition awareness and knowledge address cost, culture and behavior by:
1) creating consortiums of community based organizations.
2) forming focus groups.
3) developing programs unique to targeted communities.
4) preparing material that promotes use of new foods.
5) holding cooking classes.
6) evaluating the effectiveness of their intervention. All of these can be a part of a Medical Home that addresses the health needs of the community it serves.

Education is a *component of *rather than a *substitute for *income support and supplemental food programs. Without a foundation of opportunity, education alone is an example of the "unfreedom" of chronic poverty [38] where the least educated and empowered people in society are expected to function with the highest degree of sophistication in food selection and preparation. [39]

## Conclusion

The interaction among genetic, social, cultural and economic element should not be considered separately. The data provided suggests that the appearance of discretionary income is an economic factor closely related to the prevalence of the various forms of under- and overnutrition. No cause and effect relationship is suggested. Rather the appearance of discretionary income, essentially the availability of that last dollar earned for discretionary spending, provides an indicator of the risk for undernutrition, overnutrition, and nutrient imbalance in communities of varying levels of wealth. Working to ensure the availability of food and other necessities as incomes rise to secure levels is one part of the process of preventing the undernutrition of chronic poverty from becoming the obesity of near poverty. Our goal is to move families to the high levels of nutritional status associated with affluence using a combination of income and food support, and community based nutrition education. Ensuring an income of opportunity – secure discretionary income – is an essential element in promoting community wide nutrition.
